# Vascularised fibular graft in the management of non-union of fracture shaft of radius: a less ventured entity

**DOI:** 10.4314/gmj.v54i4.13

**Published:** 2020-12

**Authors:** Tapas K Panigrahi, Ramesh C Maharaj, Debi P Nanda

**Affiliations:** Department of Orthopaedics, Sriram Chandra Bhanja Medical College, Cuttack, Odisha, India

**Keywords:** radius fractures, fracture fixation, internal, fibula, locked dynamic compression plate

## Abstract

**Introduction:**

Non-union of the radius and ulna is a major complication of forearm fractures, accounting upto 10% of all forearm fractures. Multiple modalities are available for the treatment of non-union. Vascular grafts are a less sought-after surgical choice owing to the need of expertise and skills of surgeons. We discuss a case of gap non-union of fracture shaft radius treated with vascular fibula graft.

**Case Report:**

We describe a case of 45yr old lady with closed fracture of both bones of left forearm.

She underwent open reduction and internal fixation with 3.5 small DCP (6 hole) two days following trauma. On subsequent follow up in 6 months the radius fracture showed signs of infected non-union with osteolysis at screw sites while the ulnar side showed signs of satisfactory union. The patient underwent debridement with implant removal and osteosynthesis with vascularised fibula for gap non-union as second stage. 3 and 6 months follow up showed improvement in DASH score as well as VAS score and fair return of regular activity.

**Conclusion:**

In management of gap non-union of Shaft radius with gap (>6cm) vascularised fibular graft provides excellent functional outcome with far less donor site complications.

**Funding:**

None declared

## Introduction

Non-union rates after screw and plate fixation of diaphyseal fractures of forearm are upto 10%.^1^ Fracture gapping after 3.5-mm screw and plate application occurs because of inadequately followed principles of fixation which will prevent primary bone healing and possibly result in non-union. Furthermore, around one-third of non-union occur in the presence of a deep surgical site infection.^2^ Various treatment modalities exist which include cortico-cancellous bone graft from iliac crest, illizarov method, and vascularised and nonvascularised autologous bone graft followed by fixation with 3.5 Dynamic Compression Plate(DCP).

It has been concluded in studies that technique of bone block grafting to correct diaphyseal defects of the radius or ulna is relatively easy to perform and has a high success rate^3^ but the massive amount of graft required for an 8cm gap is associated with multiple donor sites complication.^4^ Illizarov method being cumbersome in forearm with time to recovery being long was not opted for.^5^ Free vascularised fibular grafting(FVFG) is a successful form of treatment for large bony defects and use of modern techniques of fixation does not affect the risk of non-union when compared with traditional forms of fixation.^6^ Here we discuss a case pf gap non-union of radius treated with FVFG and fixation with 3.5 locking DCP.

## Case Report

A 45yr old lady suffered from a road traffic accident and sustained a closed diaphyseal fracture of both the bones forearm on left side. She underwent Open reduction and internal fixation of both the bones after 2 days following trauma with 3.5 Small DCP(6 hole). On subsequent follow up at 6 months with her primary surgeon it was found that there was osteolysis at the radius site while the ulna showed good signs of union. She was complaining of persistent pain (VAS Score-4) and unable to do routine activities with the involved limb. We evaluated her radiographs and did routine haematological tests. The parameters suggested there is some deep tissue infection (Raised Erythrocyte Sedimentation Rate {ESR} and C-reactive protein {CRP}) but the wound was healthy with healthy scar.

**Figure 1:**
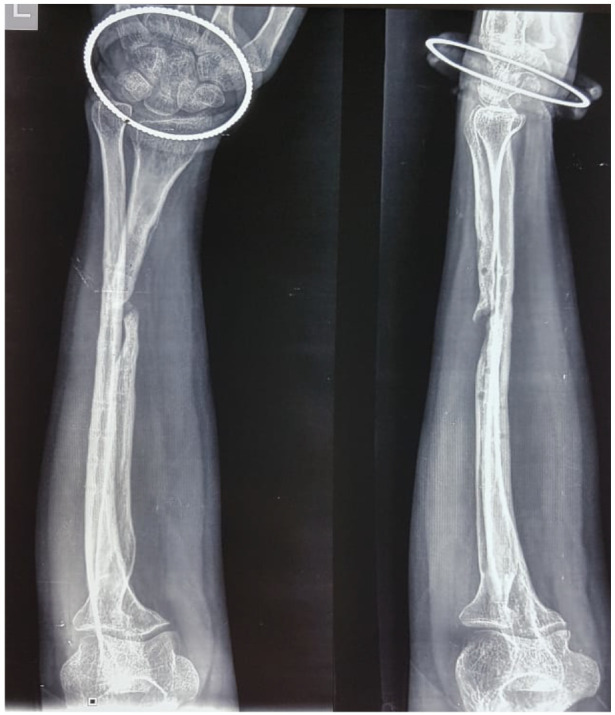
Initial preoperative radiograph

The patient was planned for staged surgery. First stage for implant removal with debridement, lavage of fracture site with culture sensitivity studies. Second stage for osteosynthesis with FVFG with plate once the acute phase reactants settled down. After a period of 2 months we again admitted the patient and did the routine investigations. As it was expected there was near normalcy of the parameters of infection like ESR, CRP. We diagnosed this as a case of gap non-union of shaft radius as evident from radiological investigation (Figure 1) and described all the treatment alternatives to the patient. After obtaining all the necessary consent we decided to do a free vascularised fibular grafting and fixation with a locking 3.5 small DCP. (12 hole)

**Figure 2:**
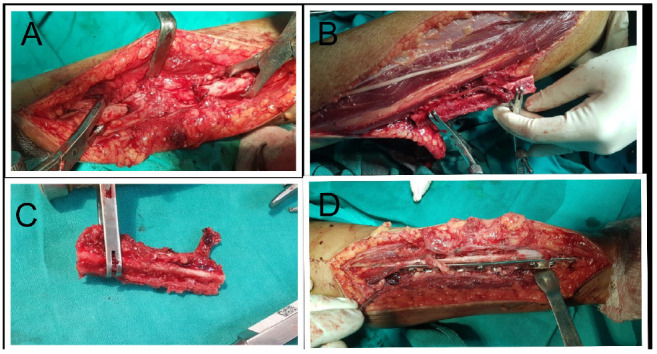
Intra operative pictures :(A) The radial gap of 8 cm. (B) Clamping and harvesting of fibular graft. (C) Final FVFG with the arterial loop. (D)Fixation with 3.5 Locking DCP.

We approached the radius through the previous incision site and freshened the atrophic ends of radius and finally found the gap to be 8cm (Figure 2A). The ipsilateral fibula was harvested in its middle 2/3^rd^ taken as a pedicle with the peroneal artery, which arises from the posterior tibial artery, 3 to 4 cm distal to its bifurcation into the anterior and posterior tibial arteries (Figure 2B, 2C). It was fixed in the recipient site with the use of a 3.5 locking Dynamic Compression Plate (Figure 2D). With the aid of a microscope and the use of 8-0 polyamide suture, the feeder artery for the graft was anastomosed to the radial artery in end to end manner. Both the donor and recipient site was closed after thorough wound irrigation and drains in situ.

Post-operative slabs were given both in upper and lower limb.

**Figure 3:**
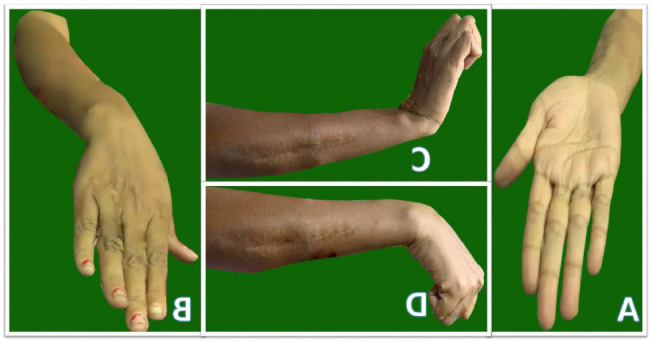
Follow up after 6 months: (A) Supination (B) Pronation (C) Extension (D)Flexion

**Figure 4:**
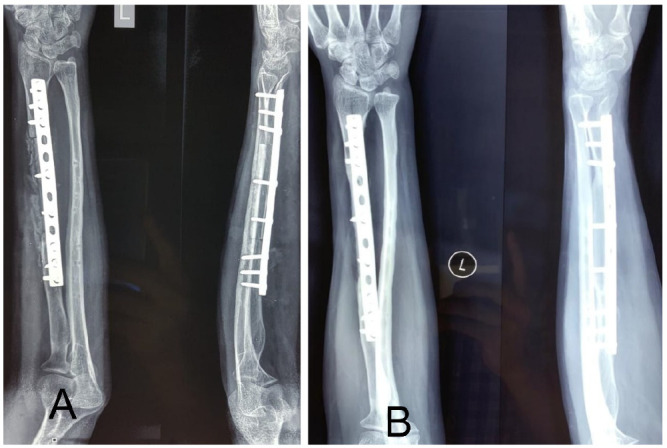
Post-operative radiographs: (A) Immediate Post op (B) 6 months follow up

The patient was followed up monthly with radiographs and serum parameters of infection. She was allowed to do free range of movement exercise for elbow and wrist but non weight lifting. At 6 months there is satisfactory movement in elbow and wrist (Figure 3) and the graft is completely healed (Figure 4).

## Discussion

Non unions of both bone of forearm is not so uncommon and among that gap non-union of any single bone is very rare. While iliac crest grafting is mostly preferred by surgeons it is associated with a lot of donor site morbidities. Other methods such as Radialisation of Ulna in Post Infective Gap Non-union Radius leads to limitation of movements due to single bone construct.^7^ In defects greater than 6–8 cm, vascularized grafts have been proposed to have significant advantages over conventional grafts.^8^ It has been recommended that the vascularized fibular graft to be used in patients who have intractable non-union that have failed to respond to conventional bone grafting or who have large bone defects (>6 cm).9 Fibula being not a principal transmitter of body weight in lower limb doesn't affect the gait or leg functions and movement.

## Conclusion

Though a procedure of expertise free vascularised fibular grafting should always be borne in mind while treating gap non unions of forearm to give satisfactory movement and function to the patient's affected limb.
